# Combined X-ray absorption and SEM–EDX spectroscopic analysis for the speciation of thorium in soil

**DOI:** 10.1038/s41598-023-32718-x

**Published:** 2023-04-11

**Authors:** Sanduni Ratnayake, Johannes Lützenkirchen, Nicolas Finck, Dieter Schild, Frank Heberling, Teba Gil-Díaz, Kathy Dardenne, Jörg Rothe, Horst Geckeis

**Affiliations:** 1 Institute for Nuclear Waste Disposal, Karlsruhe Institute of Technology, Hermann-von-Helmholtz Platz 1, 76344 Eggenstein-Leopoldshafen, Germany; 2 Institute of Applied Geosciences, Karlsruhe Institute of Technology, Adenauerring 20b, 76131 Karlsruhe, Germany

**Keywords:** Environmental sciences, Solid Earth sciences

## Abstract

Mobility and bioavailability of radionuclides in the environment strongly depend on their aqueous speciation, adsorption behavior and the solubility of relevant solid phases. In the present context, we focus on naturally occurring Th-232 at a location in central Sri Lanka presenting high background radiation levels. Four different soil samples were characterized using X-ray Absorption Spectroscopy (XAS) at the Th L_3_-edge (16.3 keV), Scanning Electron Microscopy (SEM) and Energy Dispersive X-ray (EDX) spectroscopy. X-ray Absorption Near Edge Structure (XANES) spectra are applied as a fingerprint indication for Th existing in different chemical environments. Linear combination fitting (LCF) of the Extended X-ray Absorption Fine Structure (EXAFS) data involving reference Th-monazite (phosphate) and thorianite (oxide) compounds suggested that Th is mostly present as Th-phosphate (76 ± 2%) and Th-oxide (24 ± 2%), even though minor amounts of thorite (silicate) were also detected by SEM–EDX. Further studies on selected individual particles using micro-focus X-ray Fluorescence (μ-XRF) and micro-X-ray Absorption Spectroscopy (μ-XAS) along with SEM–EDX elemental mapping provided information about the nature of Th-bearing mineral particles regarding mixed phases. This is the first study providing quantitative and XAS based speciation information on Th-mineral phases in soil samples from Sri Lanka.

## Introduction

Thorium (Th) is a naturally occurring radioactive element, which is more abundant in the Earth’s crust (i.e., ~ 8 × 10^–4^ wt.% ) than uranium (i.e., ~ 4 × 10^–4^ wt.%) and other radioactive elements^1^. In fact, the average concentration of Th in the upper continental crust is ~ 10 – 15 mg kg^-1^^2^. Naturally occurring Th is dominated by the primordial radioisotope Th-232 (half-life: 1.40 × 10^10^ years). This radioisotope contributes to the background radiation levels worldwide (i.e., Th-232 content ~ 30 Bq kg^-1^^3^), even though several regions on the planet have high background radiation areas (HBRAs). The most known cases for soil samples characterized by monazite sand deposits (rich in Th) are found in Guarapari and Meaipe in Brazil (i.e., annual average effective dose rate 1.5 mSv yr^-1^^4,5^), Yangiang in China (i.e., 5.4 mSv yr^-1^^6^), the states of Kerala and Madras in India (i.e., 15.7 mSv yr^-1^^7^), and the Nile delta in Egypt (i.e., < 0.07 mSv yr^-1^^8^). Less known HBRAs are also present in Sri Lanka, showing the highest average Th-232 concentration in soils among the identified regions in Asia (i.e., Th-232 content between 9 – 1166 Bq kg^-1^)^9^. Until now, most studies in Sri Lanka have focused on Th-rich beach deposits identified along the coastal areas of the country^10^, presenting a mean effective dose rate of 1.2 mSv yr^-1^^11^. However, little is known about the mineralogical characteristics of Th phases inland, where hotspots of Th-rich areas are also present^12^. The impact of high natural radioactivity on health of the potentially concerned population is still subject of epidemiological and biological studies. Up to now unequivocal correlations of radiation level and health issues have not been evidenced (see e.g., *Aliyu and Ramli*^13^).

The major mineral phases of Th are monazite ((Ce, La, Nd, Th)PO_4_), thorite (ThSiO_4_) and thorianite (ThO_2_)^14^. Pure forms of natural ThO_2_ and ThSiO_4_ crystals are rare due to their common association with monazite, uraninite, and other major phases. Thorium can also be present in trace amounts in mixed phases of phosphate, oxide and silicate minerals^15,16^. Notably, the possible mobility of thorium upon release from mineral phases is of interest, potentially contributing to the transport into groundwater reservoirs or the food chain^16^. Monazite exists as an accessory mineral in metamorphic and magmatic rocks. It is characterized by high stability and only slowly transforms during the degradation of the primary rock matrix. The high stability of monazite has led to discussing this phase as a candidate for the immobilization and long-term disposal of actinides^17^. Only little information is available on monazite weathering under low temperature environmental conditions, but secondary phosphate minerals such as rhabdophane or hydrated thorium oxides may form under hydrothermal conditions^18^. The nature of remaining solid phases in the soil and notably their solubility under the slightly acidic porewaters in the lateritic soil in the area of investigation will be determinant to gain a better understanding of the potential mobility of thorium^19^.

Irrespective of the prevailing redox conditions, Th exists in nature in the oxidation state + 4. The effective ionic radius of Th(IV) varies from 0.94 Å to 1.21 Å, depending on its coordination number^20^. Due to the ion's relatively large size and high charge, Th speciation may involve a large range of coordination numbers, from 4 to 15 but eightfold coordination is most common in natural systems^19,20^. Information on Th speciation can be obtained via X-ray absorption spectroscopy (XAS), which is element specific and provides molecular-scale information on the nature and number of neighboring atoms as well as on interatomic distances. Several studies on Th speciation using XAS are available in the literature while these studies have focused on (i) Th(IV) containing materials synthesized in the laboratory^21,22^, (ii) Th(IV) interaction with organic matter^23,24^, (iii) Th(IV) in natural minerals from ores^25^, and (iv) adsorption studies of Th(IV) by mineral surfaces^26–28^. To our knowledge, XAS studies of Th(IV) in natural soil samples are not currently available in the literature.

Before describing the findings of the present study, a concise summary of selected important results from a previous study on the same soil samples (Ratnayake et al., submitted), is briefly summarized in this paragraph. The soil is lateritic, and lateritic soils are acidic in nature^29^, acidic soils being defined as having pH less than 5.5 for most of the year^30^. Concerning this work, the sampled soils are reddish clayey rock material, with a surface area between 20 – 30 m^2^ g^-1^ and an average soil pH of 4.4 ± 0.2 (i.e., extremely acidic^31^). The soil organic content (TOC) is moderately high^32^, ranging between 0.69 and 1.68 g kg^-1^. Its mineralogical content was characterized using X-Ray powder Diffraction (XRD), Scanning Electron Microscopy (SEM), and Energy Dispersive X-ray (EDX) spectroscopy, X-ray Photoelectron Spectroscopy (XPS), and Attenuated Total Reflection-Fourier Transform Infrared (ATR-FTIR) spectroscopy. The XRD and ATR-FTIR results revealed that the matrix of the soil samples is dominated by kaolinite and quartz; with minor amounts of iron oxides and phosphates (< 0.2 wt.%) via standard X-ray Fluorescence (XRF). Radioactivity measurements using gamma spectrometry showed that Th-232 and its progenies have the highest contribution to the elevated background radiation. Initial laboratory scale standard Energy Dispersive XRF (ED-XRF) analysis indicated that overall Th contents are in the range of 0.06 – 0.17 wt.%. Thorium was clearly detectable and information about its chemical form was gained from detailed elemental and morphological analysis of Th-containing particles using SEM–EDX. These results showed that the chosen soil samples contain natural Th-bearing minerals suggesting that Th is present in distinct phosphate (probably monazite-type), oxide, and silicate phases.

Since the chemical speciation of the samples was not unambiguous based on those data, the present paper combines the application of XAS analyses on both bulk soil samples with standard XAS measurements and on isolated selected particles by application of micro-focused X-ray beam methods. These analyses were complemented with detailed particle-specific SEM–EDX analyses. During bulk analyses, information on coordination geometry is provided by comparing the X-ray Absorption Near-Edge Structure (XANES) region of the sample spectra with those of reference compounds. Extended X-ray Absorption Fine Structure (EXAFS) spectra yield quantitative information on the phase assemblage. XANES and EXAFS spectra are highly sensitive to the local environment and can be used as a “fingerprint” of the short-range environment of the probed element. In this sense, the chemical speciation of Th was investigated using XAS for different aliquots from the same soil samples. In all cases, the Th L_3_-edge XANES and EXAFS spectra were recorded to obtain information on the bulk speciation of the soil samples.

Since the previous study showed that the target samples are quite heterogeneous, it was anticipated that recorded bulk XAS data would correspond to the sum/average of different Th species. Consequently, additional investigations on selected particles of these samples are advisable and were performed via both SEM–EDX and XAS involving a micron-size beam footprint (µ-XAS), intending to analyze small, isolated grains from the soil samples at higher spatial resolution (SEM–EDX) or individual particles (μ-XAS). The latter was done by using a micro-focused beam to collect X-ray fluorescence data (μ-XRF) and further select points of interest for X-ray absorption spectroscopy (μ-XAS). Due to its relative simplicity and non-destructive character, µ-XRF can be used to determine elemental composition and in principle distribution (elemental mapping) for a given particle. Besides Th, lanthanides were included in the study given the fact that they are abundant in Sri Lankan soils and commonly form mixed phases with Th-bearing minerals^15^. Even though SEM–EDX has higher spatial resolution than µ-XRF, particularly in this study due to the effect of beam size vs particle sizes, µ-XRF has certain advantages over SEM–EDX regarding the elemental analysis. Particularly, this concerns the high penetration depth of X-rays, the absence of sample pre-treatments^33^, and improved detection for the analysis of heavy elements due to the absence of bremsstrahlung. The information about the composition resulting from µ-XRF comprises a larger volume of the selected particle while most of the near-surface composition is probed by SEM–EDX (i.e., only a few µm below the surface, depending on the material composition and the electron acceleration voltage). However, it is noteworthy that if the size of the particle is a few µm only, it should be taken into account that SEM–EDX may also provide information on the entire particle. Based on µ-XRF maps, additional points of interest on the individual particles were selected to obtain information on Th speciation by µ-XAS at the Th L_3_-edge.

## Methods

### Bulk soil analysis for Th-containing minerals: XANES and EXAFS analyses

For the present work, soil samples were collected from an area in central Sri Lanka with background radiation levels of 2.5 ± 1.2 µSv hr^-1^ at one-meter height above the ground, i.e., average effective dose rates of 21.6 ± 10.9 mSv yr^-1^^12^. The sampling location is the playground of a school. This renders systematic studies on the behavior of radionuclides in this area even more urgent.

The first 10 – 20 cm of four topsoil samples were collected in the Kawudupalella area in the Matale District (Sri Lanka). Aliquots of a given sample were mildly ground by hand, without damaging the particles and placed between two Kapton tapes. Reference compounds (RCs) including monazite ((Ce, La, Nd, Th)PO_4_) from Brazil and synthetic ThSiO_4_ (courtesy Dr. Stéphanie Szenknect, Institute for Separation Chemistry, Marcoule, France) were prepared in the same way as the soil samples (between Kapton tapes). A ThO_2_ reference material^21^ was prepared as a pellet. Samples and RCs were stored in a sample container to meet radioprotection requirements and transferred to the beamline. Th L_3_-edge (16.3 keV) X-ray absorption spectra were recorded at the INE-Beamline for radionuclide science^34^ at the KIT Synchrotron Light Source (KARA storage ring, Karlsruhe, Germany) with a storage ring energy of 2.5 GeV and a maximum current of 150 mA. The incoming X-ray beam was monochromatized using a pair of Ge < 422 > crystals and the energy was calibrated by assigning the first inflection point of the Th L_3_-edge XANES recorded from ThO_2_ to 16.300 keV. XAS spectra for RCs were collected in transmission mode, while fluorescence detection mode was applied for soil samples using silicon drift detectors (Vortex-ME4, Hitachi USA, for bulk sample measurements and Vortex-60EX, SII Nano Technology USA, for spatially resolved XRF/XAS measurements with µ-focused beam). Several scans were collected at room temperature and averaged to obtain adequate counting statistics.

XAS data were processed following standard procedures using the *Athena* interface to the *Ifeffit* software^35^. Further, information on the composition of the soil samples was inferred from linear combination fitting (LCF) of experimental EXAFS spectra using the spectra of the RCs. The result of LCF analysis provides direct information on the contribution of each mineral phase to the experimental data. LCF requires proper identification of the minerals present and inclusion of their respective spectra in fits of spectra of the unknown multi-mineral assemblages. Reference compounds used in LCF analysis were selected based on the major mineral phases identified in previous investigations (i.e., Th oxide, Th silicate, and Th phosphate phases; Ratnayake et al., submitted). The accuracy of this method depends on the extent to which the spectra of the chosen RCs represent the components in the unknown samples. EXAFS-LCF-analyses were performed in the k-range from 2.9 – 10.5 Å^-1^ for all samples and RCs.

### Analysis of specific Th-containing particles via SEM–EDX, μ-XRF, and μ-XAS

The soil samples were dispersed in isopropanol and dried on polished glassy carbon mounted on sample holders. The particles, dried on the carbon surfaces, were then carbon coated and subjected to SEM–EDX analysis on selected particles of interest according to the identified major mineralogy. Secondary Electron and Backscattered Electron images were recorded using an FEI Quanta 650 FEG environmental scanning electron microscope while elemental analysis in the energy dispersive mode for selected areas was performed to support the mineral characterization using a Peltier-cooled Thermo Scientific UltraDry silicon drift X-ray detector. Data were acquired and analyzed by the NORAN System7 microanalysis system, software Pathfinder version 2.8. The primary electron beam energy was 20 keV.

More detailed insight into the elemental composition along with Th-elemental distribution and the chemical state of Th was obtained from synchrotron-based μ-XRF and µ-XAS (i.e., µ-XANES) analyses. These analyses were performed using the available experimental setup at the INE-Beamline using polycapillary half-lenses to achieve an X-ray beam footprint of about 28 µm in size (FWHM of the beam intensity profile). The locations of the particles of interest within the sample holders were triangulated primarily during SEM–EDX analysis. At the beamline, initial µ-XRF scans provided the first information on elemental distribution in these regions, to relocate the particles of interest for further detailed µ-XRF analyses. An excitation energy of 17.5 keV was selected for these analyses to detect the possible presence of uranium in the samples. Subsequently, points of interest, where Th-containing phases could be identified, were selected to carry out µ-XANES analyses by recording Th L_3_-edge µ-XANES spectra.

Data were reduced and analyzed using the *PyMca* Fluorescence Toolkit^36^ and *Athena* software^35^. Comparison with the aforementioned RCs provided further information on Th-containing particle mineralogy and coordination environment. The µ-XRF, elemental mapping, and µ-XAS can be combined to characterize geochemical matrices and to identify differences in the separated Th-containing, single mineral particles. In the present study, this combination was used to determine the coordination environment of Th within isolated particles from the soil samples.

The statistical significance of the elemental distributions inside each particle (as observed in the µ-XRF elemental maps) was quantified by Spearman correlation coefficients (ρ) based on detector counts measuring the fluorescence lines from µ-XRF for the different elements. The Spearman correlation evaluates monotonic relationships whether the data are linear or not. A perfect Spearman correlation of + 1 or -1 occurs when each of the variables is a perfect monotone function of the other whilst ρ = 0 implies the absence of correlation between the variables. In the present work, the Spearman correlation coefficients are included in the discussion and both scatter plots and corresponding frequency histograms are presented in Supplementary Information online. The data treatment was performed with the statistical program R version 3.6.0^37^. All calculated correlation factors showed significant values (p-values < 0.01; see Supplementary Fig. S1, S2, and S3 online).

## Results and discussion

### Bulk XAS analysis of Th-containing minerals: Major mineral phases

**Figure 1:**
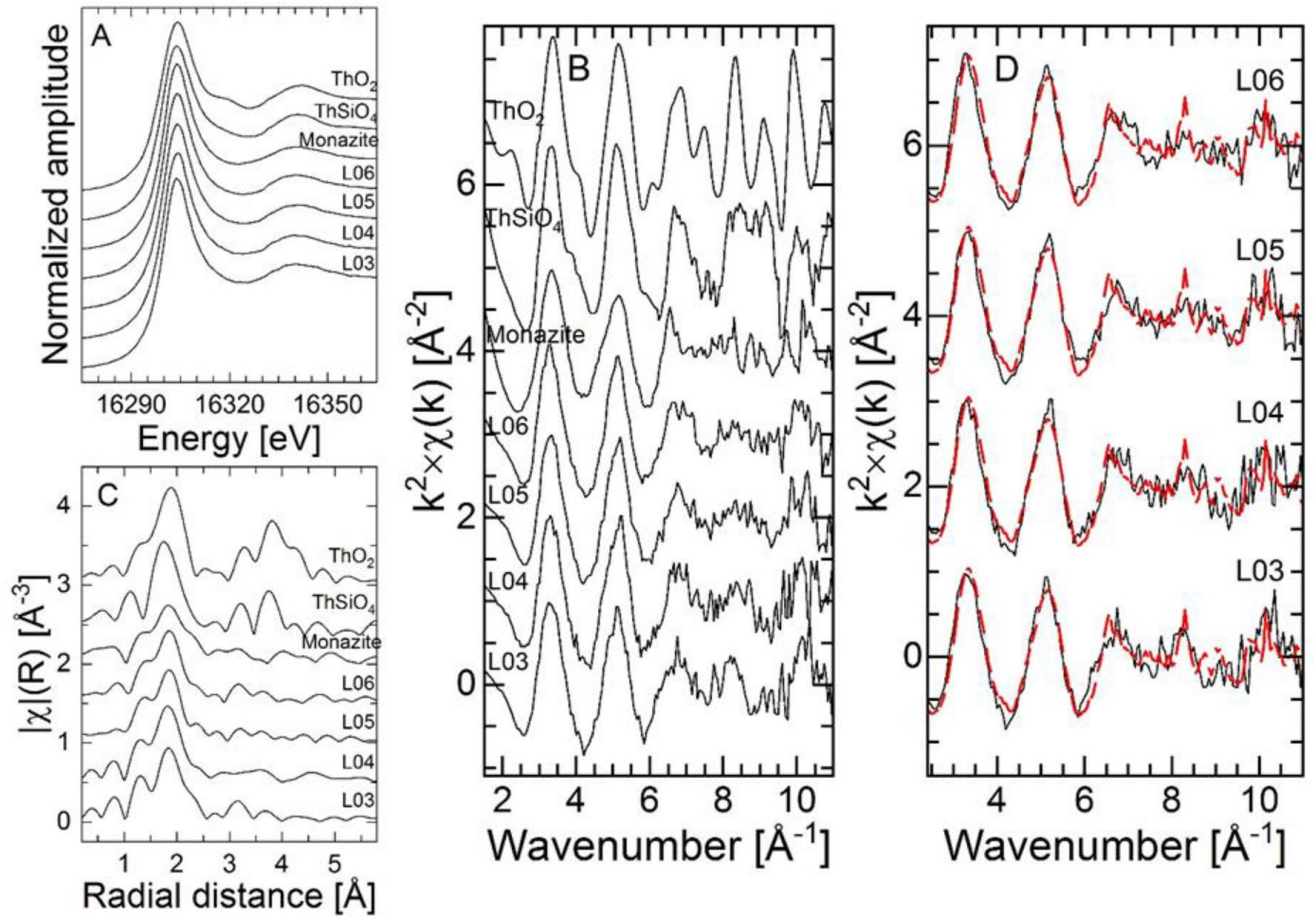
Th L_3_-edge XANES (**A**) and EXAFS spectra (**B**) with corresponding FT (**C**) of all samples and RCs (distance is uncorrected for phase shift); (**D**) experimental (solid black line) and linear combination fits (red dashed line) to the EXAFS spectra of the soil samples (fit results are shown in Table 1).

The normalized XANES spectra, k^2^-weighted EXAFS (k^2^∙χ(k)) spectra, and corresponding Fourier transformations (FT) of all soil samples and the RCs are shown in Fig. 1A, B, and C, respectively. The RCs were chosen based on the previous study, i.e., Th-silicate, Th-oxide, and Th-phosphate-containing mineral phases. From the recorded Th L_3_-edge XANES spectra (Fig. 1A), the main feature at 16,304 eV can be seen in all absorption edges. The energy positions of the absorption edges are consistent with Th in the tetravalent oxidation state in all analyzed soil samples and well in agreement with the positions of XANES of RCs, as expected for natural samples^16^. However, a second feature at 16,320 eV is more pronounced only in ThO_2_ and virtually absent otherwise. For ThO_2_ this second feature can be assigned to a solid phase exhibiting a relatively high degree of crystallinity arising from different transition probabilities due to coherently backscattered electrons. This feature is less pronounced or absent in amorphous ThO_2_ and Th(OH)_4_^38^. Therefore, the absence of the second feature in the XANES spectra could indicate that the Th-minerals in the soil do not have such high crystallinity compared to the RCs or do not contain ThO_2_.

The data in Fig. 1B (i.e., k^2^-weighed χ(k) functions) and Fig. 1C (the corresponding FT magnitudes) suggest minor variability in the short-range environment of Th among the studied soil samples, but significant differences as compared to Th within pure ThO_2_ and ThSiO_4_, and limited differences as compared to Th within monazite. Consequently, Th in the bulk soils (< 0.17 wt.%) is not present as pure single-phase ThO_2_, or ThSiO_4_, or monazite. This agrees with SEM–EDX results from the companion study, which highlighted the heterogeneity of the studied soil samples in terms of Th speciation.

All FTs (Fig. 1C)) contain one main contribution, located at R + ΔR ~ 1.85 Å, which corresponds to a phase-corrected value of about 2.4 – 2.5 Å, attributed to backscattering from oxygen atoms (Th – O) in the first coordination shell of the central absorbing Th atom. This is in accordance with reported XAS measurements on pure Th(IV) oxide/hydroxide phases^21^. At higher distances, the FT of the soil samples do not exhibit any intense FT contribution. The FT of the monazite bears similarities with that of the soil samples. The FTs of thorite and thorianite exhibit contributions from neighboring atoms at R + ΔR > 3 Å, from nearest Si/Th neighbors, in agreement with the thorite^38^ or thorianite (pure ThO_2_)^20^ crystal structures. This in turn suggests similarities between the soil samples and monazite, while the observed differences among the spectra rather purport the presence of more than one mineral phase of Th in the natural sample.

LCF treatment of the XAFS data was preferred over shell-by-shell EXAFS fitting as it was expected that soil samples contain mixtures of various Th(IV) phases including mixed phases such as solid solutions. Modeling the data using single scattering paths (i.e., Th-O, Th-Si, Th-P, Th-Th, etc.) can be complicated because of possible overlaps from different shells located at comparable distances in the host structures. The first step consisted of a statistical analysis of the data based on Principal Component Analysis (PCA)^39^ using the Athena software. Results show that the EXAFS spectrum of all soil samples can be reconstructed at more than 96% using only two principal components. In the next step, the target transformation of monazite and thorianite with two components provided best results, adding a third component provided only negligible improvement. The experimental and linear combination fits  to the EXAFS spectra of the four soil samples are shown in Fig. 1D. The linear combination fits to the experimental spectra was thus performed using only the spectrum of monazite and that of thorianite. For all samples, the experimental data could be modeled considering the same RCs with comparable proportions (Table 1). We infer from these results that monazite has the highest contribution to the experimental spectra with 76 ± 2% and thorianite the lowest contribution with 24 ± 2%. Outcomes further suggest that all samples are composed of a fairly comparable assemblage of Th-bearing mineral phases. Using thorite instead of thorianite was tested and provided systematically poorer fit quality (i.e., higher R_f_ values). Earlier findings showed the presence of ThSiO_4_ and the LCF treatment of EXAFS spectra suggest that this mineral phase is not present in significant amounts within these soil samples. In addition, samples may as well contain minor amounts of other species such as Th sorbed to minerals or bound to organic matter. This would e.g. be consistent with measurable contents of clay minerals (dominated by kaolinite), iron phases, and organic matter in these samples as identified in the previous study – phases for which Th has a high affinity. It would imply that in the soil samples Th might occur as Th-bearing mineral phases (i.e., Th-phosphate, Th-oxide) along with traces of Th retained within mineral lattices or sorbed at surfaces (i.e., iron oxides, organic matter, and clay minerals). Chemical batch extractions from a previous study (Ratnayake et al., submitted) showed that 1 – 3% of the total Th content can be extracted via single extraction procedures usually applied to identify ion exchange, carbonate, and organic matter-bound metal ions.

**Table 1:** Linear combination fitting results for the soil samples Th L_3_-edge EXAFS spectra (2.9 < k < 10.5 Å^-1^). Uncertainties are indicated in parentheses. The absolute misfit between modeled and experimental data is indicated by the R_f_ value.

Soil sample	Proportion (%)		R_f_
	Monazite	Thorianite	
L-03	78(3)	22(3)	0.21
L-04	77(3)	23(3)	0.19
L-05	76(3)	24(3)	0.15
L-06	74(3)	26((3)	0.16
Average	76 ± 2	24 ± 2	

### Spatially resolved analysis of specific Th-containing particles: SEM–EDX, µ-XRF, and µ-XANES

**Figure 2:**
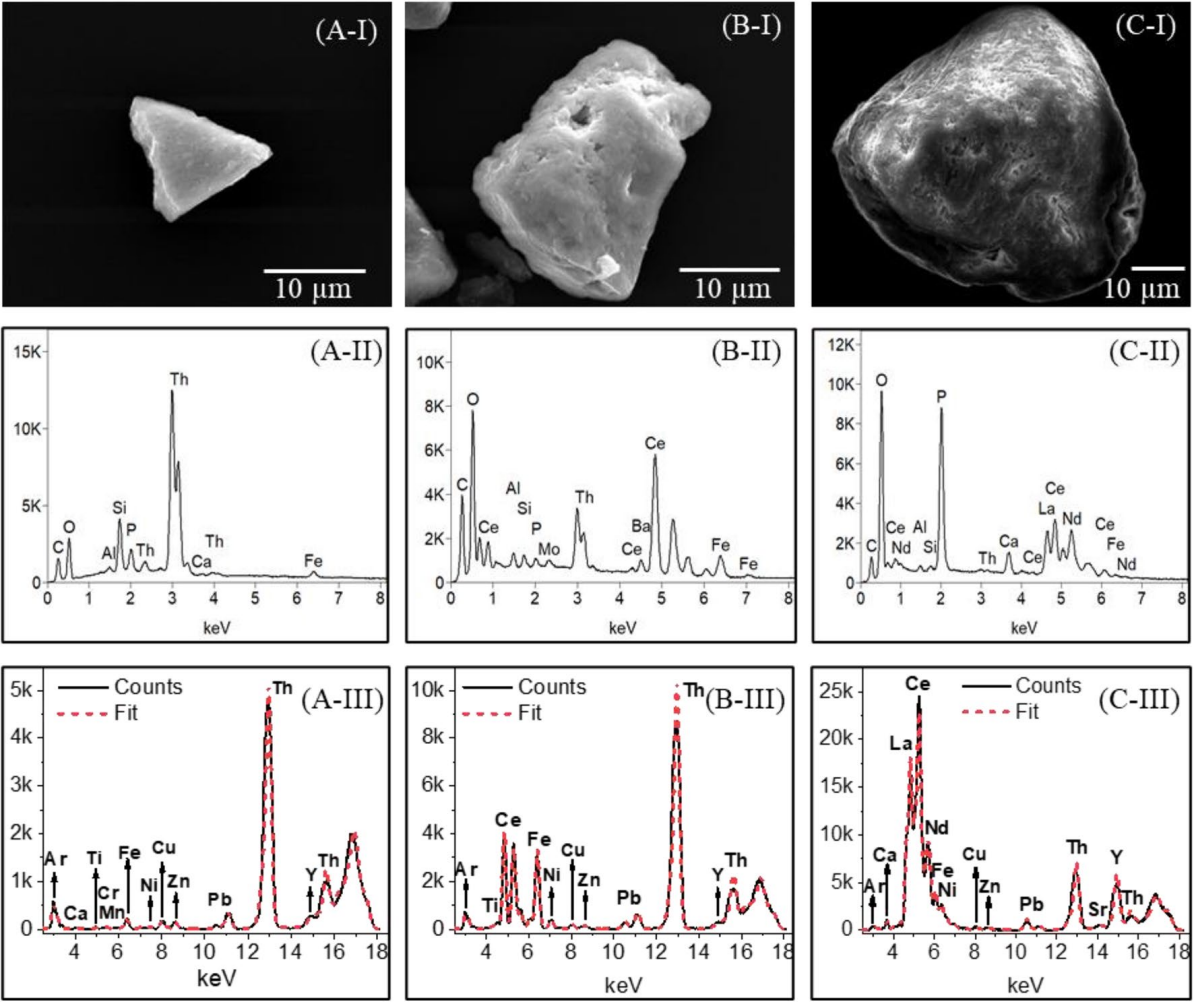
The SEM images (I), corresponding EDX (II), and µ-XRF spectra with fits (III) of selected (**A**) Th-silicate, (**B**) Th-Ce-oxide, and (**C**) Th-REE-phosphate rich particles, respectively.

Detailed information on some single particles isolated exemplarily from the four soil samples was obtained by application of SEM–EDX followed by µ-XRF and µ-XANES. According to the SEM–EDX analysis, three particles of variable size and mineralogy, identified as Th-silicate-, Th-oxide-, and Th-phosphate dominated were selected (Fig. 2A, B, and C, respectively). The corresponding weight (wt.%) and atomic (at.-%) contributions of the detected elements to each particle of interest are indicated in Table 2. SEM images of the selected particles (Fig. 2(I)) are shown together with the corresponding SEM–EDX spectra (Fig. 2(II)) and fitted µ-XRF spectra using the *PyMca* Fluorescence Toolkit to identify the elements present in the particles are displayed in Fig. 2(III). Elemental analysis using SEM–EDX was performed under a high vacuum allowing the detection of fluorescence at low energies. In contrast, analyses by µ-XRF were performed with X-rays of high energies under ambient conditions allowing the detection of fluorescence energies starting at ~ 3 keV. Since SEM–EDX is better suited to detect light elements (e.g., O, Na, Mg, Al, Si) which cannot be detected at ambient conditions using µ-XRF, the two techniques are used in a complementary fashion concerning elemental composition and the information depth of the analysis (i.e., higher resolution for surface composition in SEM–EDX vs. entire mineral composition with µ-XRF). The data show that soil samples are assemblages of particles with variable composition, and the nature of the particles agrees with the EXAFS analysis of the bulk samples.

**Table 2:** Weight and atom concentration of each element (relative %) obtained by SEM–EDX for the isolated selected particles (i.e., particle A – Th-silicate, particle B – Th-Ce-oxide, particle C – Th-REE-phosphate) and the monazite RC.

Particle	O-K	Al-K	Si-K	Fe-K	Ca-K	Ti-K	P-K	Ce-L	La-L	Nd-L	Pr-L	Th-M
Element – wt. %												
A	17.8 ± 0.3	1.2 ± 0.1	7.5 ± 0.1	1.0 ± 0.1	0.3 ± 0.0	–	2.8 ± 0.1	–	–	–	–	69.4 ± 0.4
B	30.1 ± 0.2	4.2 ± 0.1	3.2 ± 0.1	7.2 ± 0.1	–	1.6 ± 0.1	0.6 ± 0.1	37.6 ± 0.2	–	–	–	15.5 ± 0.2
C	35.4 ± 0.2	1.2 ± 0.0	1.5 ± 0.0	0.4 ± 0.1	2.2 ± 0.0	–	13.9 ± 0.1	21.4 ± 0.3	12.4 ± 0.2	8.1 ± 0.2	2.4 ± 0.3	1.1 ± 0.1
Monazite – RC*	23.0 ± 0.2	1.0 ± 0.0	1.6 ± 0.0	1.2 ± 0.0	0.2 ± 0.0	–	12.4 ± 0.1	21.8 ± 0.4	5.8 ± 0.2	14.6 ± 0.3	3.2 ± 0.5	9.5 ± 0.1
Element – Atom%												
A	60.6 ± 3.0	2.4 ± 0.5	14.5 ± 0.5	1.0 ± 0.1	0.4 ± 0.1	–	4.9 ± 0.4	–	–	–	–	16.3 ± 0.3
B	70.4 ± 1.2	5.8 ± 0.3	4.2 ± 0.3	4.8 ± 0.2	–	1.3 ± 0.1	0.7 ± 0.2	10.0 ± 0.1	–	–	–	2.8 ± 0.1
C	70.5 ± 1.4	1.5 ± 0.1	1.7 ± 0.1	0.2 ± 0.1	1.7 ± 0.1	–	14.3 ± 0.3	4.9 ± 0.2	2.8 ± 0.1	1.8 ± 0.1	0.5 ± 0.2	0.1 ± 0.0
Monazite – RC⁑	61.0 ± 1.2	1.5 ± 0.2	2.5 ± 0.1	0.9 ± 0.1	0.2 ± 0.0	–	17.0 ± 0.3	6.6 ± 0.4	1.8 ± 0.2	4.3 ± 0.3	1.0 ± 0.4	1.7 ± 0.0

*Contains Sm-L, Gd-L, U-M: 3.5, 1.6, 0.6 wt.% and ^⁑^1.0, 0.4, and 0.1 atom%, respectively.

**Figure 3:**
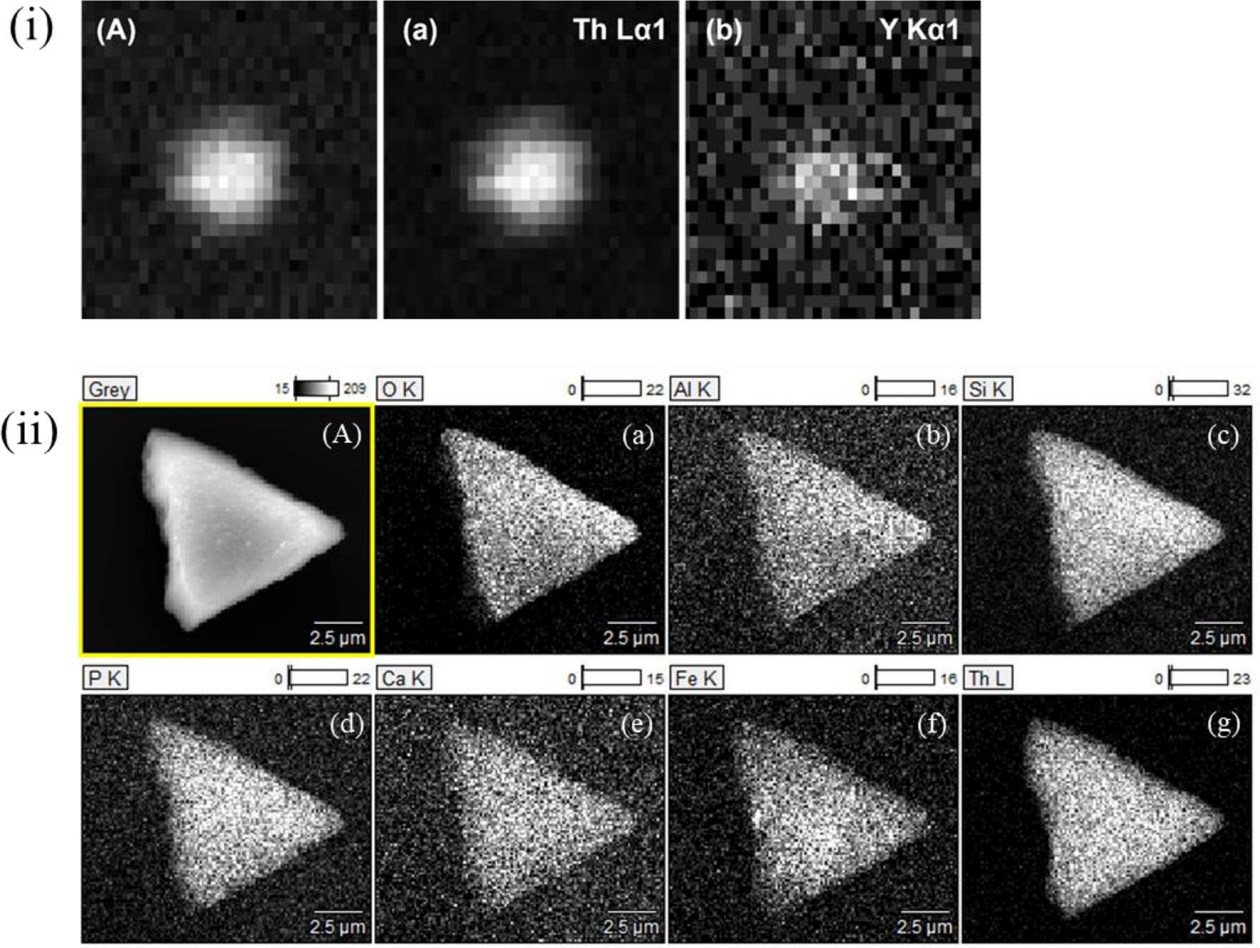
(i) µ-XRF maps of the Th-silicate particle (**A**) showing the total fluorescence and the fluorescence lines of Th (**a**) and Y (**b**) in greyscale (maps: 82.5 µm in width, 67.5 µm in height). (ii) SEM image of the Th-silicate particle (**A**) and corresponding SEM–EDX elemental maps for O, Al, Si, P, Ca, Fe, and Th (**a–g**) in counts.

The first particle to be discussed is ~ 10 µm in size (Fig. 2 (A-I)) and according to SEM–EDX contains ~ 70 wt.% of Th (Fig. 2 (A-II), Table 2). Due to the much larger beam footprint, the particle appears to have a size of ~ 30 µm based on µ-XRF mapping (Fig. 3(i)). The particle contains significant amounts of Si, compared to other major elements (Table 2), and the molar Th/Si ratio of 1.1 in the particle with the O/Si ratio of 4.2 points to the predominant existence of ThSiO_4_. Particle SEM–EDX spectra (Fig. 2(A-II)) also illustrate the presence of small amounts of Al, P, Fe, and, to a smaller extent, Ca (mapped in Fig. 3(ii)b, d, e, f). These elements could arise from trace amounts of surface-attached clays and other mineral phases, such as kaolinite and goethite. These findings are corroborated by the µ-XRF spectra (Fig. 2(A-III)), which further highlight the presence of small amounts of Pb and Y, and possibly minor levels of Ni, Cu, and Zn. Further information on the association of various elements within a given particle was obtained by recording µ-XRF maps. Figure 3(i)A depicts the total fluorescence yield, whereas Fig. 3(i)a and b show the fluorescence of selected elements recorded for the Th-silicate particle. Visual differences in µ-XRF maps (Fig. 3(i)a, b) are supported by a weak correlation between Th and Y (ρ = 0.667; see Supplementary Fig. S1 online), supporting the presence of a single major phase containing Th with smaller contributions of Y. Information on the element composition of the particle within the top µm depth, especially for the elemental distribution of the light elements, was obtained from the SEM–EDX elemental maps (Fig. 3(ii)). Both XRF (Fig. 3(i)a) and SEM–EDX (Fig. 3(ii)g) maps show homogeneous Th distribution across the particle. Heterogeneities in the Y distribution are not apparent via SEM–EDX. This might be due to the low Y-content in the sample which is close to or below the detection limit of SEM–EDX. Another possibility is that Y is mainly located below a Th silicate surface layer. The SEM–EDX maps indicate the existence of a uniform Th silicate phase with Si and O being uniformly distributed all over the particle (Fig. 3(ii)a, c).

**Figure 4:**
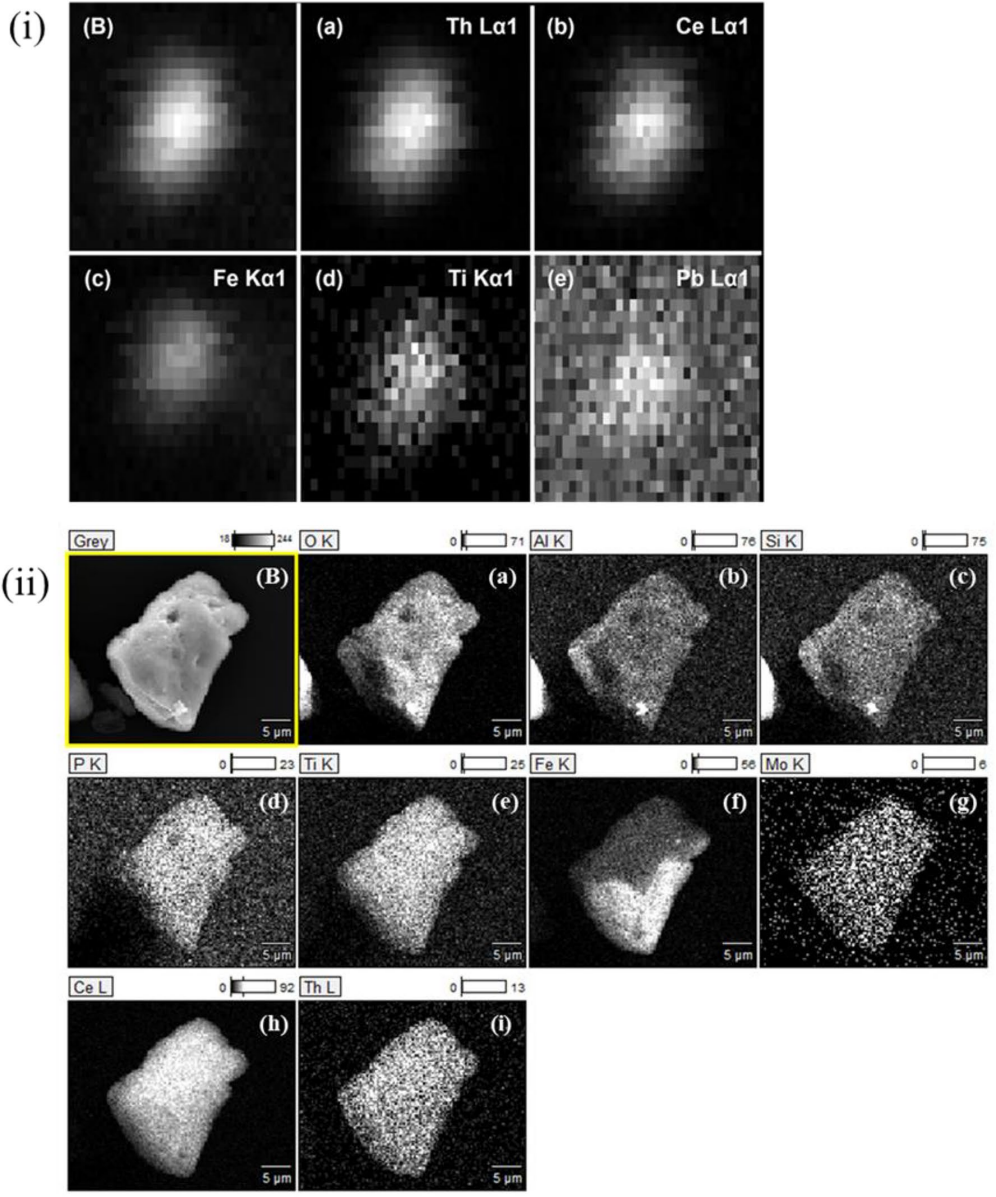
(i) µ-XRF maps of the Th-Ce-oxide particle (**B**) showing the total fluorescence and the fluorescence lines of Th (**a**), Ce (**b**), Fe (**c**), Ti (**d**), and Pb (**e**) in greyscale (maps: 107.5 µm in width, 55 µm in height). (ii) SEM image of particle (**B**) and SEM–EDX elemental maps for O, Al, Si, P, Ti, Fe, Mo, Ce, and Th (**a–i**) of the Th-Ce-oxide particle in counts.

The second particle studied (i.e., presented as a Th-oxide-containing particle) is ~ 25 µm in size, as derived from SEM (Fig. 2(B-I)), and has high Th (~ 15 wt.%) and Ce (~ 38 wt.%) contents with relatively small amounts of Fe, Al, and Si (Fig. 2(B-II), Table 2). This particle further contains Pb and possibly low levels of Cu and Ni (Fig. 2(B-III)). The molar ratio of 5.7 from O:(Th + Ce) indicates the presence of different oxidic phases in these samples. Concerning the presence of phosphates, Fe-oxides, and silicates, the O:(Th + Ce) ratio is too high for the presence of only Th/CeO_2_. The SEM–EDX analysis would rather point to some kind of Th/Ce(OH)_4_ or Th/CeO(OH)_2_. This would also be more consistent with the XANES spectra, and therefore, we conclude that the particle is dominated by (hydr)oxides, presumably Th-oxide or hydroxides mixed with Ce-oxide/hydroxide as the major component. This is further confirmed by the distribution of Th and Ce in the fluorescence maps (Fig. 4(i)B and 4(i)a, b), presenting a significant correlation (i.e., ρ = 0.970; see Supplementary Fig. S2online). In fact, literature provides evidence for (Th, Ce)O_x_(OH)_y_ solid solutions owing to the comparable ionic radii^40,41^ of Th(IV) and Ce(IV). SEM–EDX maps are shown in Fig. 4(ii)B and 4(ii)a-i. Particularly, Fig. 4(ii)f and i show an inhomogeneous distribution of Fe compared to Th, i.e., higher density areas towards the lower corner, suggesting iron oxide phases that are slightly depleted in Th, attached to the (Th, Ce)O_2_ particle covering a part of the grain. The seemingly homogeneous Fe-distribution in the XRF maps (Fig. 4(i) a, c) and significant correlation with Th (i.e., ρ = 0.806; see Supplementary Fig. S2 online) are due to the absence of adequate spatial resolution. A small clay mineral flake was for instance identified (Fig. 4(ii) b and c) on the bottom corner of the particle. Relatively consistent surface distribution of Ce can be seen in the SEM–EDX map (Fig. 4(ii)h), matching that of O and to a lesser extent to traces of Si and Al despite the mapping shadow effect, and associated clay fractions (Fig. 4(ii)a-c). The slightly weaker intensities of Th and Ce signals in the area where the Fe signal is enhanced could be interpreted as the Th/Ce-oxide particle being covered by iron oxide. Both Ce and Th are related to a small extent (0.65 < ρ < 0.85; see Supplementary Fig. S2 online) to traces of Ti and Pb (Fig. 4(i)d, e), indicating that the correlation analysis mostly reflects counting statistics. In summary, the collected data show that the natural Th-oxide particles are not pure ThO_2_ (thorianite) but presumably consist of a (Ce, Th)oxyhydroxide solid-solution covered by accessory clay minerals and Fe-oxide phases, including traces of P, Ti, Mo, and potentially also Ce (Fig. 4(ii)d, e, g, and h).

**Figure 5:**
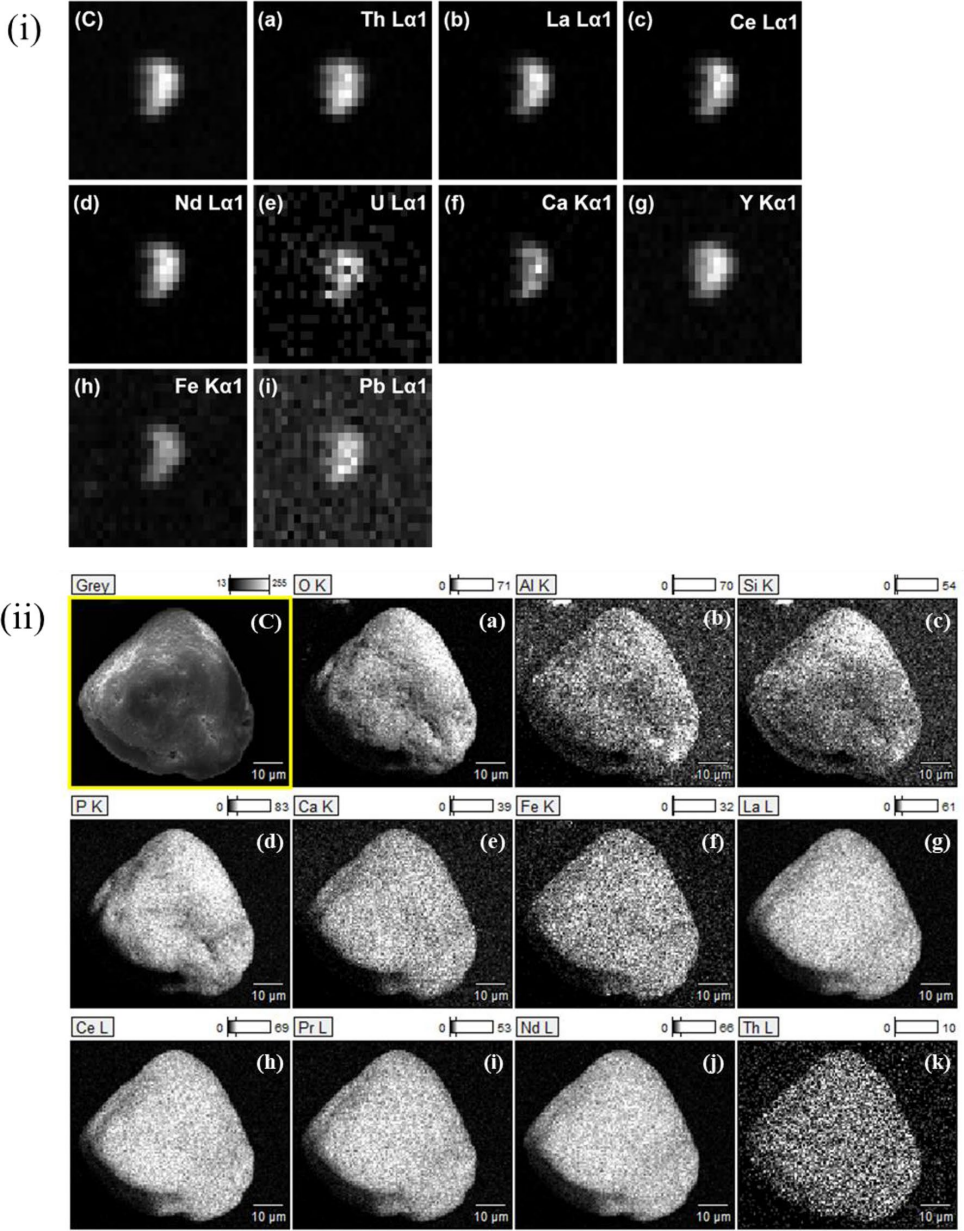
(i) µ-XRF maps of the Th-REE-phosphate particle (**C**) showing the total fluorescence and the fluorescence lines of Th (**a**), La (**b**), Ce (**c**), Nd (**d**), U (**e**), Ca (**f**), Y (**g**), Fe (**h**), and Pb (**i**) in greyscale (maps: 280 µm in width, 183 µm in height). (ii) SEM image of particle **C** and SEM–EDX elemental maps for O, Al, Si, P, Ca, Fe, La, Ce, Pr, Nd, and Th (**a–k**) of the Th-REE-phosphate particle in counts.

Finally, the ~ 50 µm sized particle (i.e., identified as a Th-phosphate-containing particle, depicted in Fig. 2(C-I)) is found to have lower Th content (~ 1 wt.%), according to SEM–EDX, compared to the two previously discussed particles, but contains significant amounts of P and considerable amounts of light REEs (Table 2). The total REE (La, Ce, Nd, Pr) content amounts to ~ 44 wt.% (Fig. 2(C-II), Table 2), comparable to ~ 45 wt.% in the RC-monazite. Hence, soil particle C is depleted in Th (stoichiometric composition: (Ce_0.43_, La_0.25_, Nd_0.16_, Pr_0.04_, Th_0.01_, Ca_0.10_)(P_0.98_, Si_0.02_)O_4_), compared to the RC monazite from Brazil (stoichiometric composition: (Ce_0.41_, La_0.11_, Nd_0.27_, Pr_0.06_, Th_0.14_, Ca_0.01_)(P_0.96_, Si_0.04_)O_4_). The high P content and the absence of significant amounts of Al and Si based on the SEM–EDX spectra (Fig. 2(C-II)) suggest that the particle indeed represents a phosphate phase. The result of the µ-XRF measurements (Fig. 2(C-III)) further indicates that this particle contains more elements than the two other studied particles (c.f. the fluorescence maps in Fig. 5(i)C and 5(ii)a-i). The iron content is low but measurable (< 1%) and exhibits the highest correlations with the analyzed light REEs and Ca (i.e., ρ > 0.92; see Supplementary Fig. S3online). Despite the low Th concentrations in this particle, Th distribution is clearly correlated to those of the REEs (i.e., ρ > 0.75; see Supplementary Fig. S3online), though the highest correlations of Th are found with Y and Pb (i.e., ρ > 0.87; see Supplementary Fig. S3online). *Berger* et al.^7^ stated that rhabdophane could also be a relevant secondary phase formed during monazite weathering. Rhabdophane is depleted in Th as compared to monazite, which would explain the low Th content of the particle. Additionally, Th, Pb, Ca, and U show positive skewness of the data distribution (see Supplementary Fig. S3 online). All of these observations suggest that monazite-type minerals may exist in a variety of compositions, in accordance with EXAFS observations. This observation is further confirmed by the work of *Seydoux-Guillaume* et al.^42^ who studied low-temperature alteration of Sri Lankan monazite samples (exact locality is unknown). We also observed REEs in mixed phases, as the SEM–EDX maps (Fig. 5(ii)C and 5(ii)a-k) for the REEs and P suggest a uniform distribution of each of these elements over the particle (Fig. 5(ii)d and g-j). A uniform distribution of Th over the particle is suggested by the EDX map (Fig. 5(ii)k). Figure 5(ii)b and c could point to the presence of Si/Al (clay minerals) particles associated with the main phosphate phase. To some extent, the data sets again suggest the presence of clay minerals and Ca-/Fe phases suggesting potential correlation with lanthanides and actinides within the particle and/or at surface.

Summarizing these results, we have shown that the combination of different focused and spatially resolved techniques such as µ-XRF (i.e., providing the bulk composition of individual particles) and SEM–EDX mapping (i.e., giving potentially the composition of both surfaces and a few μm-depth penetration layers) provides important insight into the composition and heterogeneity of single mineral grains in natural soil samples on the µm scale. The aim of all these measurements was not only to identify the heterogeneous nature of the sample (already clear from SEM–EDX analysis), but also to look for possible accessory phases like iron oxides which have coprecipitated or adsorbed Th or lanthanides. All the extensive spectroscopic studies show that particle heterogeneity has an impact on spectra, for instance in XANES. Although it is possible to identify the accessory iron oxide and clay minerals, a clear assignment of direct Th or REE binding to those accessory minerals or coprecipitated and adsorbed Th, Ln forms could not be found. Overall, the data confirm the presence of distinct, Th-containing mineral phases inside the soil samples under study while highlighting the mineral phase heterogeneity in particles even within a given sample.

### Potential applicability of particle-selected spectra (µ-XANES) as representative standards for bulk XANES reconstruction

**Figure 6:**
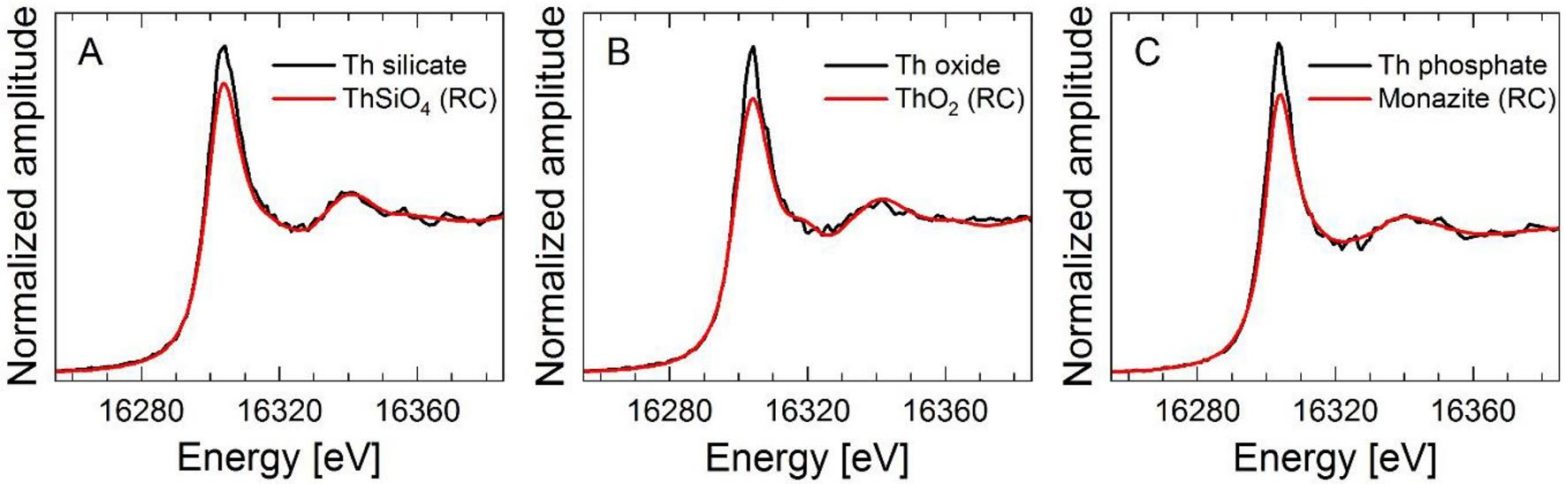
Micro-X-ray Absorption spectra of (**A**) Th-silicate, (**B**) Th-oxide, and (**C**) Th-phosphate rich particles, respectively, with the overlaid XANES spectrum recorded for the ThSiO_4_ (**A**), ThO_2_ (**B**), and monazite (**C**) reference compound.

Additional Th L_3_-edge µ-XANES spectra were collected for particles A, B, and C (Fig. 6 A, B, and C, respectively). The energy position of the XANES white line in all analyzed particles is identical to that in the bulk soil samples. This is not surprising as the oxidation state of Th in environmental samples is always + 4. It is obvious that the X-ray absorption spectra of these particles closely follow those of the corresponding RCs even though their XAS signals are quite noisy. Theoretically, the identified particles could be used as local RC, to fit the bulk soil spectra in both XANES and EXAFS regions, which should lead to more conclusive results compared to the applied synthetic Th-silicate/oxide RCs and the natural monazite RC. This was tested for the XANES region because only µ-XANES spectra could be recorded for the selected particles. Unfortunately, the EXAFS region could not be measured during µ-XAS data acquisition because fluorescence intensity was not sufficient. In addition, the backfitting of the µ-XANES spectra was hampered by the relatively high intensities of the white lines from the identified Th-containing particles. This effect is potentially related to differences in composition between the natural particle from Sri Lanka and laboratory synthesized (ThO_2_ and ThSiO_4_) and natural monazite RC from Brazil and/or to the difference in particle sizes when collecting the spectra, for instance, the monazite RC particle was ~ 40 times bigger than particle C. Consequently, the spectra of individual particles that we used in this study cannot be utilized as representative standards adding the facts that the composition of selected particles may not be representative of the average composition of the entire sample, and the entire sample may also contain an additional Th binding environment not present in the selected particles (e.g., surface sorbed species). These observations further highlight the challenges in characterizing the speciation of Th in natural samples, composed of multiple mineral phases compared to studies on pure samples. Subsequently, they call for future efforts with a smaller beam footprint and a confocal setup to precisely single out the contribution of individual particles from adhering accessory matrix components.

## Conclusions

The combination of scanning electron microscopy and X-ray spectroscopic techniques has proven to be powerful in characterizing naturally occurring Th-rich soil samples from Sri Lanka at various scales. X-ray absorption spectroscopy of soil samples at the Th L_3_-edge suggests that the soil contains a mixture of various Th-bearing minerals, mainly as (hydr)oxides and phosphates, and in lower amounts as silicates. Individual particle analysis by µ-XANES and µ-XRF as well as corresponding elemental maps, supported by statistical analyses, provide insight into the association of Th with other elements at the scale of single particles. Complementary information from SEM–EDX allows the identification of the elemental distributions in the particle surface layer, which may differ from the underlying mineral characteristics, indicating particle coverage by other mineral phases such as clay minerals and iron oxides (i.e., as seen for particle B). The combined approach is one of a kind regarding characterization of Th speciation in soil via XAS techniques. This work underpins results with non-negligible implications for terrestrial samples, i.e., showing differences between bulk and surface compositions of individual soil particles of ~ 10–50 µm size (i.e., in the silt–clay grain size range). Overall, these results provide original information on environmental Th solid phases that may react differently to weathering agents (i.e., given their small size and high surface area), potentially having an impact on the release, mobility and bioavailability of Th in the target area. Without this work, complementary studies in the direction of Th mobility and reactivity from natural soils would lack the in-depth understanding of Th phases provided here. That is, this work lays the foundation to understand mobilization mechanisms, predict the environmental fate and assess the radiological risk of radioactive Th. Clearly, despite the low contribution of Th-minerals to the overall mineralogy of the soil (< 0.17 wt.%), further work is needed to evaluate the potential mobilization of Th during weathering (i.e., during rainfall and under the biological influence) to better understand its potential fate and/or accumulation in aquatic and terrestrial systems.

## Supplementary Information

Supplementary Information.

## Data Availability

Data will be made available on request to Sanduni Ratnayake (sanduni.ratnayake@kit.edu).

## References

[1] Krauskopf, K. B. & Bird, D. K. Surface chemistry: the solution-mineral interface. In: MG-HI ED (ed.) *Earth Sciences and Geology Series*, McGraw-Hill International Editions edn, New York, 135–163 (1995).

[2] Rudnick, R. L. & Gao, S. Composition of the continental crust. In: *Treatise on Geochemistry* vol. 3 (eds Holland, H.D. & Turekian, K.K.) 1–64. 10.1016/b0-08-043751-6/03016-4 (Elsevier-Pergamon, Oxford, 2003).

[3] UNSCEAR, Sources and Effects of Ionizing Radiation. 2000, United Nations: New York.

[4] Fujinami, N., Koga, T. & Morishima, H. External exposure rates from terrestrial radiation at Guarapari and Meaipe in Brazil. IRPA-10 Proceedings of the 10th International Congress of the International Radiation Protection Association on harmonization of radiation, human life and the ecosystem, (p.1v). Japan: Japan Health Physics Society (2000).

[5] Costa de Moura, J., Wallfass, C.M. & Bossew, P. Health hazards of radioactive sands along the coast of Espirito Santo/Brazil. *Neues Jahrbuch für Geologie und Paläontologie-Abhandlungen Band 225 Heft 1* 127–136. 10.1127/njgpa/225/2002/127 (2002).

[6] Wei, L. *et al.* Epidemiological investigation in high background radiation areas of Yangjiang, China. In *High Levels of Natural Radiation* (ed Durrani, S.A.) 618. (International Atomic Energy Agency (IAEA), IAEA, 1993).

[7] Sohrabi M (1998). The state-of-the-art on worldwide studies in some environments with elevated naturally occurring radioactive materials (NORM). Appl. Radiat. Isot..

[8] Badawy WM (2018). Environmental radioactivity of soils and sediments: Egyptian sector of the Nile valley. Isot. Environ. Health Stud..

[9] Nalaka DS (2013). Measuring radon and thoron levels in Sri Lanka. Adv. Mat. Res..

[10] Herath, M.M.J.W. Beach Mineral Sands in Sri Lanka. Occurrence, Global Trends and Current Issues, Geological Survey and Mines Bureau, Colombo (2008).

[11] Warnakulasuriya T (2020). Background radiation levels near a mineral sand mining factory in Sri Lanka: Correlation of radiation measurements with micronuclei frequency. Radiat. Prot. Dosim..

[12] Baseline Radioactivity Data, Sri Lanka Atomic Energy Board, Colombo, https://aeb.gov.lk/soil/ (Accessed on 10 October 2017).

[13] Aliyu AS, Ramli AT (2015). The world's high background natural radiation areas (HBNRAs) revisited: A broad overview of the dosimetric, epidemiological and radiobiological issues. Radiat. Meas..

[14] René, M. Nature, sources, resources, and production of Thorium. In *Descriptive inorganic chemistry researches of metal compounds* 201–212. 10.5772/intechopen.68304 (IntechOpen, London,2017).

[15] Tennakone K (2011). Thorium minerals in Sri Lanka, history of radioactivity and thorium as a future energy source: A compendium to commemorate the International Year of Chemistry 2011. J. Natl. Sci. Found. Sri Lanka..

[16] Langmuir D, Herman JS (1980). The mobility of thorium in natural waters at low temperatures. Geochim. Cosmochim. Acta..

[17] Dacheux N, Clavier N, Podor R (2013). Monazite as a promising long-term radioactive waste matrix: Benefits of high-structural flexibility and chemical durability. Am. Mineral..

[18] Berger A, Gnos E, Janots E, Fernandez A, Giese J (2008). Formation and composition of rhabdophane, bastnäsite and hydrated thorium minerals during alteration: Implications for geochronology and low-temperature processes. Chem. Geol..

[19] Östhols, E. Some processes affecting the mobility of thorium in natural ground waters. Doctoral thesis, Royal Institute of Technology (KTH), Stockholm, Sweden (1994).

[20] Tutson CD, Gorden AEV (2017). Thorium coordination: A comprehensive review based on coordination number. Coord. Chem. Rev..

[21] Rothe J, Denecke MA, Neck V, Müller R, Kim JI (2002). XAFS investigations of the structure of aqueous Thorium(IV) species, colloids, and soil thorium(IV) oxides/hydroxide. Inorg. Chem..

[22] Hector AL, Levason W (2005). Periodates of tetravalent titanium, zirconium, hafnium, and thorium: Synthesis, characterization and EXAFS study. Eur. J. Inorg. Chem..

[23] Denecke MA, Bublitz D, Kim JI, Moll H, Farkes I (1999). EXAFS investigation of the interaction of hafnium and thorium with humic acid and Bio-Rex 70. J. Synchrotron Radiat..

[24] Hennig, C. *et al.* Identification of hexanuclear Actinide (IV) carboxylates with Thorium, Uranium, and Neptunium by EXAFS spectroscopy. *J. Phys.: Conf. Ser.* 430. 10.1088/1742-6596/430/1/012116 (2013).

[25] Luo Y (2011). Crystal chemistry of Th in fluorapatite. Am. Mineral..

[26] Östhols E, Maceau A, Farges F, Charlet L (1997). Adsorption of thorium on amorphous silica: An EXAFS study. J. Colloid Interface Sci..

[27] Hu B (2017). New insights into Th (IV) speciation on sepiolite: evidence for EXAFS and modeling investigation. Chem. Eng. J..

[28] Dähn, R., Scheidegger, A. M., Manceau, A., Baeyens, B. & Bradbury, M. H. Local structure of Th complexes on montmorillonite clay mineral determined by extended X-ray absorption fine structure (EXAFS) spectroscopy. In: Biannual Report 1999/2000, *Project-Group ESRF-Beamline (ROBL-CRG),* FZR-322, 33–38 (2001).

[29] Ranasinghe RMSC, Werellagama DRIB, Weerasooriya R (2014). Arsenite removal from drinking water using naturally available laterite in Sri Lanka. Engineer: J. Inst. Eng. Sri Lanka..

[30] FAO. Acid Soils. FAO Soils Portal 2019, http://www.fao.org/soils-portal/soil-management/management-of-someproblem-soils/acid-soils/en/ (Accessed on 22 July 2019).

[31] NRCS. Examination and Description of Soil Profiles. National Soil Survey Handbook 2019, http://www.nrcs.usda.gov/wps/portal/nrcs/detail/soils/ref/?cid=nrcs142p2_054242 (Accessed on 03 June 2020).

[32] Kaplan D, Serkiz S (2001). Quantification of thorium and uranium sorption to contaminated sediments. J. Radioanal. Nucl. Ch..

[33] Komatani S, Aoyama T, Nakazawa T, Tsuji K (2013). Comparison of SEM-EDS, micro-XRF and confocal micro-XRF for electric device analysis. Surf. Sci. Nanotechnol..

[34] Rothe J (2012). The INE-beamline for actinide science at ANKA. Rev. Sci. Instrum..

[35] Ravel B, Newville M (2005). Athena, artemis, hephaestus: data analysis for X-ray absorption spectroscopy using IFEFFIT. J. Synchrotron Radiat..

[36] Solé VA, Papillon E, Cotte M, Walter PH, Susini JA (2007). Multiplatform code for the analysis of energy-dispersive X-ray fluorescence spectra. Spectrochim. Acta B: At. Spectrosc..

[37] R. Core Team, R: A language and environment for statistical computing. http://www.R-project.org (Accessed on 25 May 2020).

[38] Galanzew, J., Electronic Structure Studies of Th Systems by high Energy X-Ray Spectroscopy and Computational Methods, in Institute for Nuclear Waste Disposal, Karlsruher Institut für Technologie (2018).

[39] Wasserman SR, Allen PG, Shuh DK, Bucher JJ, Edelstein NM (1999). EXAFS and principal component analysis: a new shell game. J. Synchrotron. Radiat..

[40] Shannon RD, Prewitt CT (1969). Effective ionic radii in oxides and fluorides. Acta Crystal..

[41] Shannon RD (1976). Revised effective ionic radii and systematic studies of interatomic distances in halides and chalcogenides. Acta Crystal..

[42] Seydoux-Guillaume A-M (2012). Low-temperature alteration of monazite: Fluid mediated coupled dissolution–precipitation, irradiation damage, and disturbance of the U–Pb and Th–Pb chronometers. Chem. Geol..

