# Supplementary material for: Combined X-ray absorption and SEM–EDX spectroscopic analysis for the speciation of thorium in soil
Source: Sci Rep. 2023 Apr 11;13:5877. doi: 10.1038/s41598-023-32718-x (PMC10090180; doi:10.1038/s41598-023-32718-x)
Supplement: Supplementary file 1 — Supplementary Information. [file 41598_2023_32718_MOESM1_ESM.pdf]

Supplementary Information (SI)

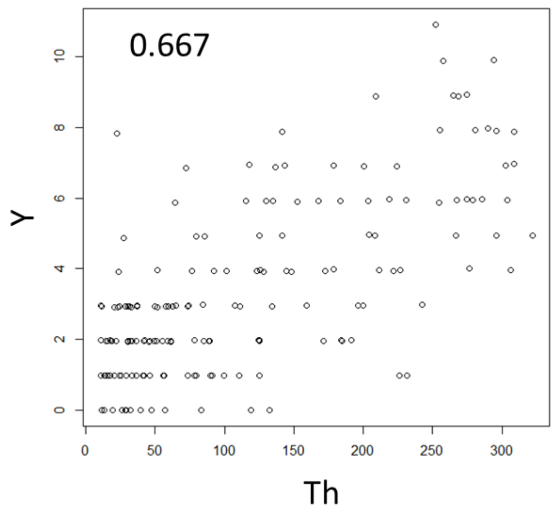

Figure S1: X-ray fluorescence scatter plot and Spearman correlation coefficients between fluorescence counts of Th and Y for the Th-silicate particle (A)

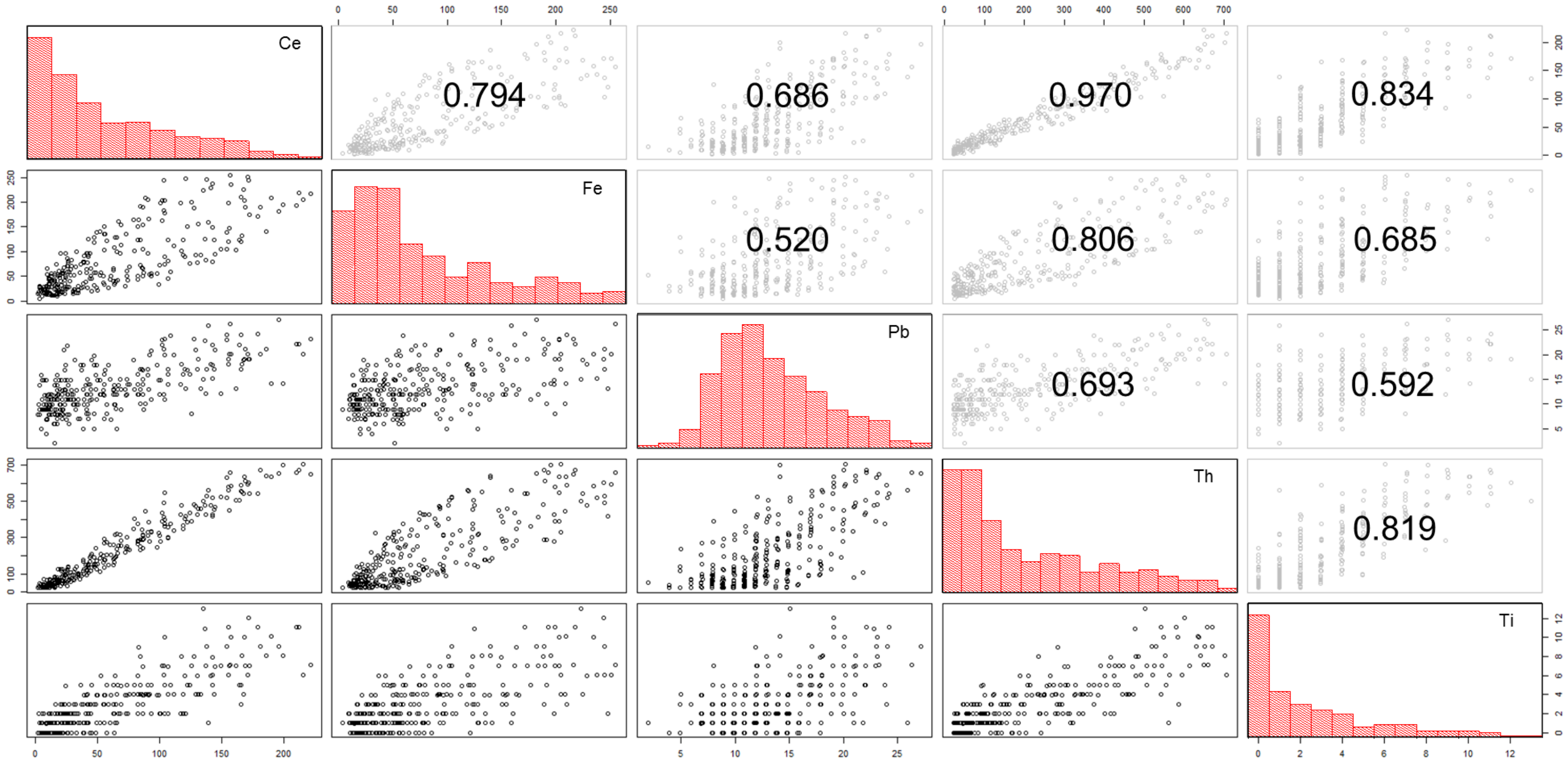

Figure S2: X-ray fluorescence scatter plots, histograms and Spearman correlation coefficients between fluorescence counts of Ce, Fe, Pb, Th, and Ti for the Th-oxide particle (B)

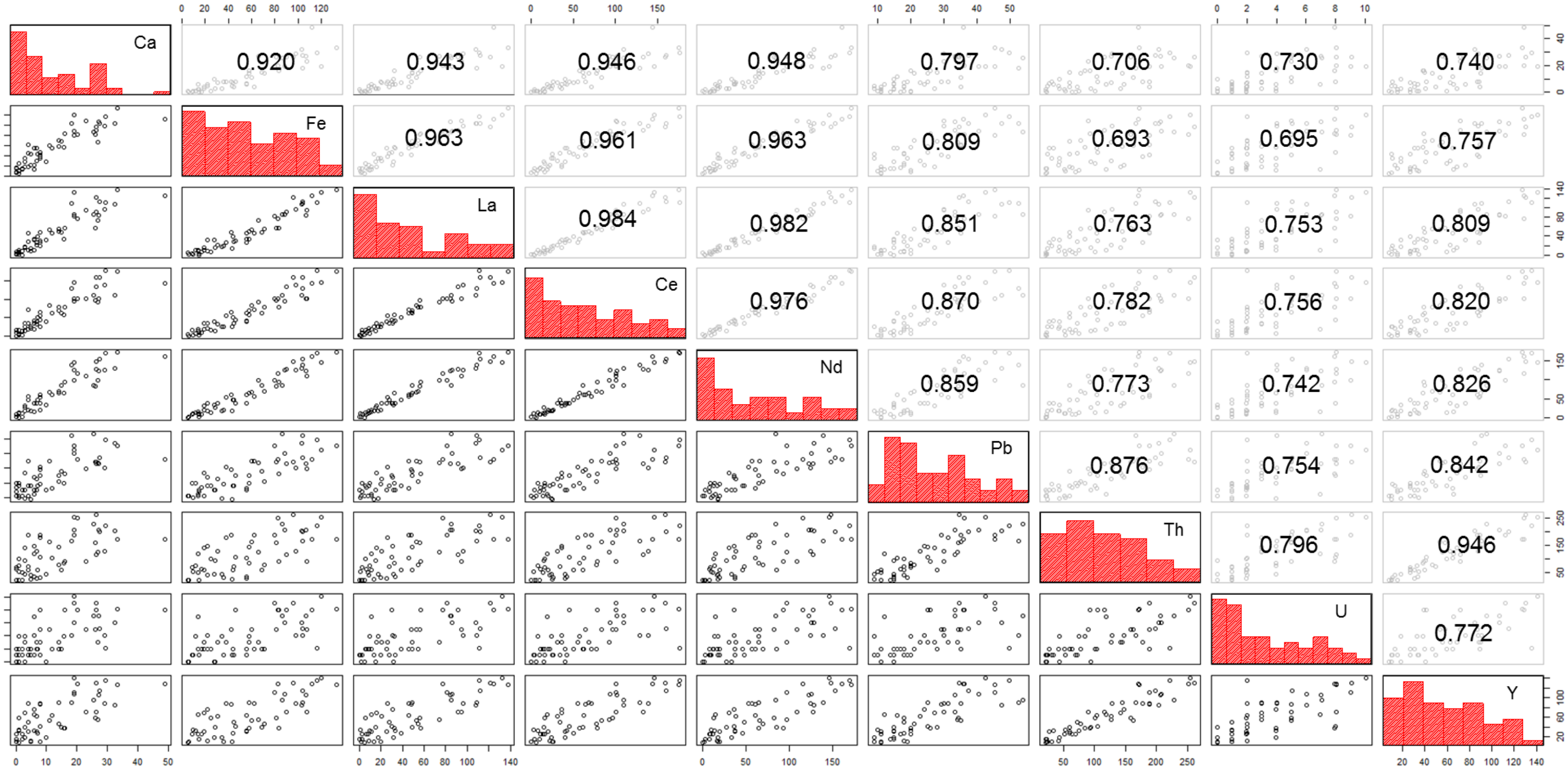

Figure S3: X-ray fluorescence scatter plots, histograms and Spearman correlation coefficients between fluorescence counts of Ca, Fe, La, Nd, Pb, Th, U, and Y for the Th-phosphate particle (C)
